# Performance analysis for similarity data fusion model for enabling time series indexing in internet of things applications

**DOI:** 10.7717/peerj-cs.500

**Published:** 2021-05-20

**Authors:** Mina Younan, Essam H. Houssein, Mohamed Elhoseny, Abd El-mageid Ali

**Affiliations:** 1 Faculty of Computers and Information, Minia University, Minia, Egypt; 2 Faculty of Computers and Information, Mansoura University, Mansoura, Egypt; 3 Department of Computer Science, American University in the Emirates, Emirates, United Arab Emirates

**Keywords:** Internet of things, Data reduction, DTW, Searching, Time series, Clustering, Indexing

## Abstract

The Internet of Things (IoT) has penetrating all things and objects around us giving them the ability to interact with the Internet, i.e., things become Smart Things (SThs). As a result, SThs produce massive real-time data (i.e., big IoT data). Smartness of IoT applications bases mainly on services such as automatic control, events handling, and decision making. Consumers of the IoT services are not only human users, but also SThs. Consequently, the potential of IoT applications relies on supporting services such as searching, retrieving, mining, analyzing, and sharing real-time data. For enhancing search service in the IoT, our previous work presents a promising solution, called Cluster Representative (ClRe), for indexing similar SThs in IoT applications. ClRe algorithms could reduce similar indexing by O(K − 1), where K is number of Time Series (TS) in a cluster. Multiple extensions for ClRe algorithms were presented in another work for enhancing accuracy of indexed data. In this theme, this paper studies performance analysis of ClRe algorithms, proposes two novel execution methods: (a) Linear execution (LE) and (b) Pair-merge execution (PME), and studies sorting impact on TS execution for enhancing similarity rate for some ClRe extensions. The proposed execution methods are evaluated with real examples and proved using Szeged-weather dataset on ClRe 3.0 and its extensions; where they produce representatives with higher similarities compared to the other extensions. Evaluation results indicate that PME could improve performance of ClRe 3.0 by = 20.5%, ClRe 3.1 by = 17.7%, and ClRe 3.2 by = 6.4% in average.

## Introduction

The Internet of things (IoT) paradigm allows things and objects (e.g., animals, bulbs, cars, persons, etc.) to speak on the Internet by enabling them to express their states. The lower layer of the IoT, called the sensing or perception layer, contains things attached with smart devices such as sensors and actuators to be managed through the Internet, such things are defined as Smart Things (SThs). They are the key elements for building IoT applications (Younan et al., 2020b). Entities of Interest (EoI) is another concept that is used to express an object state but bases on multiple sensors or SThs (e.g., room state). So, the IoT is the Internet of SThs and EoIs. Social IoT (SIoT) (Fu et al., 2019) is a new paradigm that highlights the phrase “*always connected*”. In general, main layers of the IoT are (a) perception layer, where SThs are located, (b) network layer, where base-stations or gateways communicate with SThs, (c) cloud layer, where sensed data is stored, and (d) application layer, where users can act with and visualize things states (Younan et al., 2020b).

Main features about SThs and data in the IoT could be summarized in the following points, but not limited: (a) heterogeneous, due to variety of SThs connected to the Internet, (b) huge (IHS markit; Howell, 2017), due to massive SThs integration and real time data production, and (c) dynamic, where SThs states are changed and monitored frequently in the real-time. Thus, IoT data could be described by five Vs (volume, value, variety, velocity, and veracity) as listed in ur Rehman et al. (2017). An extensive survey on the big data in IoT applications was presented in Ge, Bangui & Buhnova (2018) for discussing re-usability and selection of big data technologies in different IoT applications.

Because IoT applications are in increasing fashion, it is essential to support and enhance the future IoT with services like search and discovery for handling such deluge of SThs and data. Main root causes of IoT big data could be summarized as follows (Younan et al., 2020b): (a) increasing SThs integration for achieving high accuracy for sensed features (events), for handling fault tolerance, and for enabling ubiquitous sensing, (b) isolation of IoT applications due to some security issues, i.e., installing private SThs instead of sharing resources with threaten applications, (c) not supporting SThs as consumers for services such as searching and discovery. Due to dynamicity of IoT data, data analysis become challenging task (Younan et al., 2020a), traditional indexing methods are not convenient for IoT applications. For IoT search engines (IoTSEs), indexes became out-of-date once they are created or updated (Gonzalez-Gil, Martnez & Truong, 2020). Main challenges for searching in the IoT applications were summarized in Younan et al. (2020b), Gonzalez-Gil, Martnez & Truong (2020) as follows: (a) heterogeneity of resources (b) none standardization for annotating data, (c) privacy and resources limitations, and (d) large-scale of IoT applications. Mobility and latency are encountered as resources discovery challenges (Khalil et al., 2020).

For handling big data resulting due to mobility of SThs, Limkar & Jha (2019) propose a parallel construction for R-Tree using Apache Spark for handling geo-spatial indexing. Recent researches try to invent new methods and algorithms for handling big data in the IoT. In this theme, Edge/fog computing presents a promising solution for handling massive data exchange between SThs and the cloud (Amadeo et al., 2018; Dautov & Distefano, 2017; Bellavista et al., 2019), where cloud services and capabilities become near from the perception layer in the IoT. For the same purpose, a distributed search at the edge computing was presented in Noguchi et al. (2018). Also, crawlers are implemented on the fog layer for filtering and detecting malicious SThs integration (Albdour, Manaseer & Sharieh, 2020). Dyser (Ostermaier et al., 2010) search engine and WoTSF (Younan, Khattab & Bahgat, 2016) framework suggest indexing summary of SThs data in form of prediction models. Range query (Fathy et al., 2016) is a special type of search engines that indexes range of SThs readings, because they slightly differ. This type of information is called semi-dynamic. According to Fathy et al. (2016), distributed indexes are recommended for indexing IoT data. Studies (Younan et al., 2020a) and (Tran et al., 2017) recommend and present solutions for balancing data indexing and updating.

Because search service has a precious impact on IoT applications as studied in Younan et al. (2020a), the previous research works (Younan et al., 2020a) and (Younan et al., 2020c) focus on similarity data fusion for enabling balanced indexing for SThs Time Series (TS). Novel communication techniques were presented in Younan et al. (2020b), Papageorgiou, Cheng & Kovacs (2015), and Al-Qurabat, Abou Jaoude & Idrees (2019) to reduce redundancy of readings and data transmission to save power consumption. A novel similarity data fusion model based Dynamic Time Warping (DTW) (Salvador & Chan, 2007), called **Cl**uster **Re**presentation (ClRe) were presented in Younan et al. (2020a) and improved in Younan et al. (2020c) to generate representatives with higher similarities *∀* TS. Novelty of this method is indexing single STh dataset (i.e., cluster representative) per each cluster of TS, which captures most of all SThs behaviors (i.e., sensing patterns). Contributions of this paper are summarized as follows:

- Presenting a brief overview on ClRe algorithms and their extensions for analyzing their performance in terms of run-time complexity, representative accuracy, and representative length.
- Proposing a new method for parallel execution, called Pair-merge execution (PME).
- Testing all permutations of datasets in each cluster to study impact of sorting datasets by dissimilarity value on ClRe performance.

The remaining of this paper is arranged as follows. Related works that recommend searching, crawling, and balanced indexing for IoT resources (SThs) are discussed in the next section. “ClRe Algorithm” presents a brief overview for ClRe algorithms and their extensions (proposed in the previous works). “The Proposed Execution Methods for ClRe Algorithm” proposes two execution methods for enhancing ClRe performance for indexing clusters representatives with higher accuracy. “Discussion and Performance Analysis” discusses performance evaluation results of ClRe running in the proposed modes. Conclusion and future work are presented in “Conclusion”.

## Related Work

This section discusses related work in terms of recent research works that highlight recommendations on searching and indexing IoT resources, followed by recent search engines and frameworks that enable IoT search. Finally, recent studies on balancing IoT data indexing.

Multiple studies such as Younan et al. (2020b), Tran et al. (2017) and Barnaghi & Sheth (2016) review and summarize searching challenges and requirements in the IoT. Research works (Tran et al., 2017) and (Pattar et al., 2018) highlight recommendations for an indexing mechanism for balancing data indexing and refreshing. In Khalil et al. (2020), discovery techniques in the IoT were reviewed and classified into data-based and object-based. IoT resources require special type of crawlers (Baldassarre et al., 2019). In Gonzalez-Gil, Martnez & Truong (2020), IoTCrawler project was presented as a secure IoT search engine for searching in dynamic and pervasive resources. Locating IoT crawlers at the fog layer could aide in securing IoT applications by detecting malicious actions of SThs (Albdour, Manaseer & Sharieh, 2020; Younan et al., 2020a). Some IoT search engines are presented for purpose such as Shodan (2013), which indexes SThs’ banners and similar sensor search (Truong, Römer & Chen, 2012). IoT-SVKSearch (Ding, Chen & Yang, 2014) and Dyser (Ostermaier et al., 2010) present static and dynamic query search. Table 1 presents sample of recent IoT search engines, frameworks, and crawlers.

**Table 1:** Examples for IoT search engines (IoTSEs) prototypes and frameworks.

Year	References	IoT Search engine, Framework, or Crawler
2007	Grosky et al. (2007)	SenseWeb
2008	Tan et al. (2008)	Microsearch
2010	Wang, Tan & Li (2009)	Snoogle
2010	Ostermaier et al. (2010)	Dyser
2013	Shodan (2013)	Shodhan
2014	Ding, Chen & Yang (2014)	IoT-SVKSearch
2015	Lunardi et al. (2015)	COBASEN
2016	Younan, Khattab & Bahgat (2016)	WoTSF
2016	Kamilaris, Yumusak & Ali (2016)	WOTS2E
2016	Shemshadi, Sheng & Qin (2016)	ThingSeek
2018	Baldassarre et al. (2019)	CDS
2019	Tang et al. (2019)	SMPKR
2020	Gonzalez-Gil, Martnez & Truong (2020)	IoTCrawler

As mentioned earlier data fusion is a promising solution for handling real-time big data in the IoT (Akbar et al., 2018). The wisdom is to reduce redundancy in the lower layer, where sensors are integrated (i.e., on SThs level). Liu, Song & Liu (2020) implemented data aggregation for improving data gathering in Wireless Sensor Networks (WSNs) on the level of sensors to increase network life-time, their proposed model bases on data generation and transmission cycles (i.e., transmission bases on wake up cycles). This level of data aggregation corresponds to the perception layer in the IoT. For For the same purpose, Liu, Song & Liu (2020), Huang et al. (2020) propose SPS-IUTO and ACMC schemes, respectively for data gathering. In the previous work (Younan et al., 2020a), threshold data and time are used statically and semi-dynamically for deciding on data transmission, but the difference is that these models integrate stability and prediction of SThs readings in the decision. The second proposed model for data aggregation in Younan et al. (2020a, 2020c) is done in the fog layer—on the gateway level (i.e., STh datasets are already gathered)—for each cluster to build higher level indexes as proposed in Younan, Khattab & Bahgat (2016).

Ramachandran et al. (2018) reduce search space by enabling a cluster-based search. Similarly, this work and previous works (Younan et al., 2020a, 2020c) suppose that SThs are grouped/clustered to be indexed. Indexing clusters representatives not only allows data reduction, but also keeps indexes as up-to-date as possible. Moreover, enabling search in cluster representatives enhances search engines performance by saving time consumed in crawling, indexing, retrieving, and ranking. Liu et al. (2018) focus on indexing the most valuable attributes. DTW (Salvador & Chan, 2007) has multiple applications in this them, For instance, in Kocyan et al. (2013) and Maschi et al. (2018) it is used for identifying repeating episodes (i.e., assessing similarities between patterns). According to Vaughan & Gabrys (2016), it is used for combining time series datasets. Thus, this paper proposes new execution methods for improving ClRe performance to index similar SThs resources.

## ClRe Algorithm

This section presents a brief overview for Cluster Representative (ClRe) algorithms and their extensions. ClRe algorithms are presented in the previous works (Younan et al., 2020a, 2020c).

In Younan et al. (2020a), a new communication architecture is presented for reducing data transmission between SThs and their gateways saving resources consumption (e.g., battery) to increase SThs life-time. Moreover, a novel similarity data fusion model for reducing indexed data in the IoT is proposed. This model bases on indexing clusters representatives for similar SThs. In IoT applications, SThs are clustered by type and location (i.e., SThs measure the same phenomenon in the same environment). Validity and consistency of clustering is assessed by DTW, Silhouette is another metric for effective clustering (Kalpakis, Gada & Puttagunta, 2001). For the same cluster, dissimilarity between datasets is measured by the DTW. Resulting clusters representatives have to combine/aggregate SThs datasets in a form that capture almost of SThs’ behaviors. So, dissimilarity value of the resulting cluster representative is the main criteria for assessing and improving ClRe algorithm performance. Table 2 shows a brief summary for performance analysis for all ClRe extensions; where K is number of datasets in the cluster and n is maximum dataset length (maximum number of STh readings). All extensions are compared based on their run-time complexity, memory complexity required to generate cluster representative, and resulting representative length and average dissimilarity between cluster representative and all datasets in the cluster. Average dissimilarity value and length are ranked in ascending order (1:5), i.e., ‘1’ means the least dissimilarity value and the least length, while ‘5’ the highest dissimilarity value and the highest length.

**Table 2:** Performance analysis for ClRe algorithm extensions.

Algorithm	Run-time complexity	Memory complexity	Length rank	Dissimilarity rank
ClRe 1.0	*O*(*K*^2^*n*^2^)	*O*(*K*^2^)	2	3
ClRe 1.1	*O*(*K*^2^*n*)	*O*(*K*^2^)	2	3
ClRe 2.0	*O*(*Kn*)	*O*(*n*)	5	4
ClRe 3.0	*O*(*Kn*^2^)	*O*(*n*)	3	2
ClRe 3.1	*O*(*Kn*^2^)	*O*(*n*)	4	1
ClRe 3.2	*O*(*Kn*^2^)	*ω*(*n*)	1	5

The main idea of ClRe 1.0 and its extension (ClRe 1.1), is to select higher similarity dataset to be the cluster representative, then assessing similarity to *K* dataset between datasets and each other consumes running time of *O*(*K*^2^ *DTW*). Because ClRe 1.0 uses DTW then its running time complexity is *O*(*K*^2^*n*^2^), while ClRe 1.1 consumes *O*(*K*^2^*n*); where it uses FastDTW. The two extensions consume the same memory complexity to store resulting dissimilarity values between datasets and the other in the cluster. ClRe 2.0 gets the average of each corresponding times (readings in the same time for all SThs), thus its running time is *O*(*Kn*) and resulting representative dataset consumes *O*(*n*) memory space. ClRe 3.0 and its extensions (ClRe 3.1 and ClRe 3.2) base on handling warped items after getting the warped path between each pair of datasets for capturing SThs behavior in a new one called inner/temporary representative, but getting the accurate warped path relies on assessing similarity using DTW, thus their running time consumes *O*(*Kn*^2^), the required memory for storing resulting dataset is of range of maximum dataset length.

In brief, promising solutions for balancing time series indexing in the IoT based on priority of some criteria are as follows. ClRe 3.1 for capturing most of SThs behavior in the cluster, where it has the higher average similarity. ClRe 1.1, where it has balanced running time, average similarity, and dataset length. ClRe 3.2, for indexing only common behavior for all SThs in the cluster, which means that the resulting representative will has less number of items (*ω*(n)).

## The Proposed Execution Methods for clRe Algorithm

As listed earlier, one of the main reasons for assessing, measuring, or monitoring certain phenomenon (e.g., *CO*_2_ level) using multiple SThs of the same type, is certainty of readings. In such cases, one of those SThs is selected to be indexed (i.e., filtering SThs), while in other cases all SThs behaviors are required to be expressed as a single STh (i.e., aggregation). Thus, the term accuracy in this theme means having least dissimilarity score *∀* TS in the cluster. ClRe algorithms (versions 1.0, 2.0, and 3.0) are presented in Younan et al. (2020a), based on their performance analysis, ClRe 1.0, which bases on DTW and its extension ClRe 1.1, which bases on FastDTW, have the minimum running time, *O*(*Kn*^2^) and *O*(*Kn*), respectively, while ClRe 3.0 and its extension ClRe 3.1 have the higher accuracy (i.e., less dissimilarity values for TS in their clusters). For enabling similar SThs indexing, main targets proposed in Younan et al. (2020a), Papageorgiou, Cheng & Kovacs (2015), and in this paper are: (a) reducing index size, which is achieved by indexing only one STh (TS) per cluster—and (b) increasing accuracy of indexed data, which is achieved as well by decreasing dissimilarity values of indexed clusters representatives.

**Figure 1:**
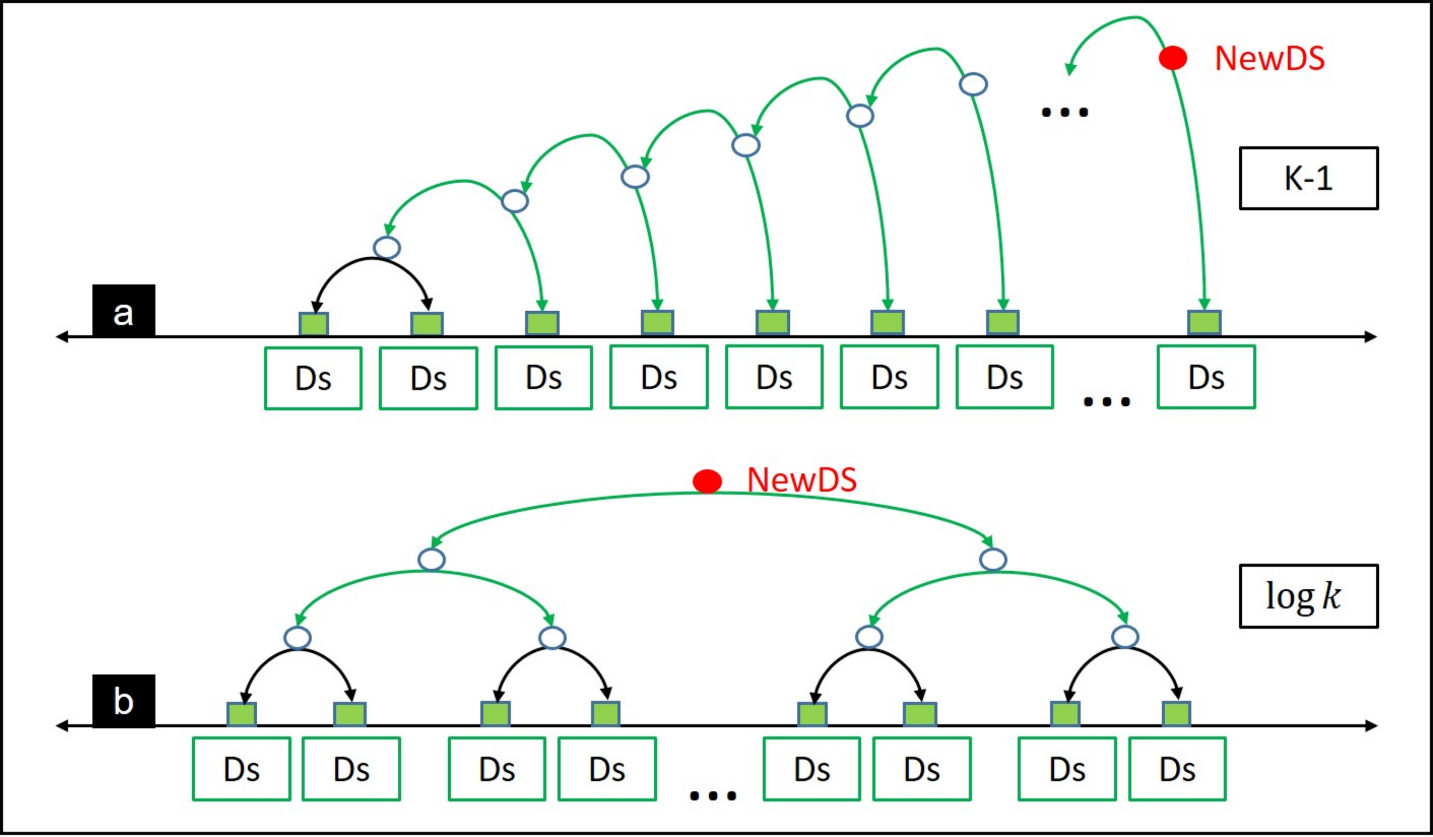
Execution methods for ClRe algorithm: (A) Linear and (B) Pair-merge.

Because reducing TS of the cluster representative could deflect or deviate main behaviors of SThs, the main goal of this paper is to increase accuracy of indexed data by decreasing dissimilarity values of the clusters representatives. This could be achieved if the resulting dataset (i.e., cluster representative) captures almost behaviors of SThs in the cluster. Based on the experimental results discussed in the next section, ClRe 3.1 is the promising solution in this theme. Enhancing running time of this extension is another goal in addition to its accuracy. So this section proposes two methods for execution, as shown in Fig. 1: (a) sequential execution (trivial method), called linear representative and (b) parallel execution, called pair-merge representative.

Similar to merge sort algorithm, a representative dataset will be generated for each pair of TS in the lower level, and recursively on the resulting datasets representatives at each higher level to the root (final representative). The proposed execution methods are shown in Algorithms 1 and 2, respectively.

**Algorithm 1:** Linear execution: generate accumulative representation for cluster datasets.

**Result**: Cluster Representative (NewDS)
**Input**: Cluster datasets: ClrDSList
**Output**: New Dataset: NewDS
// Initialization.
1 NewDS=ClrDSList[0] // initialize with the first dataset in the cluster
// Call ClRe extension for building accumulative representation.
2 for *i in range (1:len(ClrDSList))* **do**
3 X= NewDS
4 Y= ClrDSList[i]
5 NewDS= ClRe Ex(X,Y)
6 **end**
7 Return NewDS

**Algorithm 2:** Pair-merge execution: generate representative for each pair at each level.

**Result**: Cluster Representative (NewDS)
**Input**: Cluster datasets: ClrDSList
**Output**: New Dataset: ClrDSList[0]
// Call ClRe extension for building inner representatives.
1 **while** *len(ClrDSList)¿1:* **do**
2 odd=len(lset)%2
3 NewList=[]
4 count=0
5 **for** *i in range (0,len(ClrDSList)-odd,2):* **do**
6 X=ClrDSList[i]
7 Y=ClrDSList[i+1]
8 NewList.append(ClRe V(X,Y))
9 **end**
10 **if** *(odd==1):* **then**
11 X=ClrDSList[len(ClrDSList)-1]
12 Y=NewList[len(NewList)-1]
13 NewList[len(NewList)-1]= ClRe V(X,Y)
14 **end**
15 ClrDSList=NewList
16 **end**
17 Return ClrDSList[0]

Analysis of running time complexity consumed by the proposed execution methods are indicated as follows, where ClRe V represents any extension of ClRe 3.0 (e.g., ClRe 3.1). As mentioned earlier (Table 2), ClRe 3.0 and its extensions consume *O*(*Kn*^2^).

- **Linear execution method (LE):** this method is the trivial execution method, it starts by calling ClRe V to generate a representative between the accumulative representation and current dataset in the cluster, so run time complexity of ClRe V will not affected as indicated in Eq. (1).

(1) $$\begin{array}{cc} T(n) & =\sum_{i=1}^{K}ClRe(AccRep,List[i])\\ & =(K-1)(C_{1}(n^{2})+C_{2}(N))\\ & =O(Kn^{2}) \end{array}$$

where AccRep is the accumulative representation dataset, List[i] is the *i*^*th*^ TS in the cluster, and (*C*_1_(*n*^2^) + *C*_2_(*N*)) is the running time consumed by ClRe V.

- **Pair-merge execution method (PME)**: this method starts at level (*log K*) by calling ClRe V between each pair, running time consumed at this level is indicated by Eq. (2), also it could be clarified as shown in Eq. (3) as a summation of calls at each level.

(2) $$\begin{array}{cc} T(n) & =\sum_{j=log(K),-1}^{0}\sum_{i=0,2}^{2^{j}}ClRe(List_{j}[i],List_{j}[i+1]) \end{array}$$

(3) $$\begin{array}{cc} T(n) & =(\frac{K}{2}+\frac{K}{4}+...+1)(C_{1}(n^{2})+C_{2}(N))\\ & =O(Kn^{2}) \end{array}$$

From these equations, the two methods the same running time complexity. The two methods generate that same number of inner representatives datasets (K − 1 datasets) for getting the final cluster representative dataset, but the second method (pair-merge) could reduce running time from *O*(*Kn*^2^) to *O*(*n*^2^ *log K*), and outperforms linear representatives in similarity rates. Cons and pros of the two methods are summarized in Table 3. However resulting representative length in range of maximum length of datasets in the cluster but it is larger than resulting representative length in sequential execution.

**Table 3:** A comparison between the proposed execution methods (LE and PME).

Criteria	Linear execution (LE)	Pair-merge execution (PME)
Main features	• Sequential execution.	• Pair execution.
	• Accumulative building for final representative at each step.	• Temporal representative for each pair.
	• One representative per iteration.	• Accumulative representative at each level (sub-tree)
Run-time complexity	• *O*(*Kn*^2^).	• *O*(*Kn*^2^).
	• No parallel execution	• Parallel: *O*(*n*^2^ *log*_2_(*K*))
Pros	• Less memory at each iteration (only one dataset of length N, where n < N < 2n).	• Allow parallel execution.
	• Resulting representative < Pair-Merge representative length.	• Throwaway representative.
		• Generates *K*/2 representatives with less dissimilarities form original datasets.
Cons	• Only sequential execution.	• At the level (*log K*), it requires (*log K*) memory spaces, each of length *N*, where n < N < 2n for representing each pair.
	• Average dissimilarity < Pair-merge dissimilarity.	• Resulting dataset length > Linear method.

## Discussion and Performance Analysis

In this section, ClRe 3.0 and its extensions (ClRe 3.1 and ClRe 3.2) are implemented using the two proposed methods (LE and PME). Evaluation is done using real examples in the first subsection and using real dataset (Szeged-weather (Budincsevity, 2016)) in the second subsection. Performance analysis is measured ∀ TS permutations into two folds; first fold is for assessing dissimilarity score in terms of; (a) Min: minimum dissimilarity value, (b) Max: maximum dissimilarity value, (c) Avg: average dissimilarity value, (d) 50^*th*^ Perc: 50^*th*^ percentile dissimilarity value, and (e) Range: dissimilarity range value (maximum value-minimum value). The second fold is for assessing resulting representative length (number of readings or items) in terms of; (a) Min: minimum number of items, (b) Max: maximum number of items, (c) Avg: average number of items, and (d) Range (maximum length–minimum length). All permutations are tested to clarify sorting impact on ClRe performance.

### Real example

This subsection tests ClRe extensions running in LE and PME modes on four clusters formed from datasets shown in Table 4 as follows; CLS_A: {a:c}, CLS_B: {a:d}, CLS_C: {a:e}, and CLS_D: {a:f}. Datasets of the real example simulate set of similar sensors (SThs) that measure or sense the same phenomenon (i.e., SThs in the same cluster), where column ’items’ represents 6 datasets corresponding to 6 sensors (SThs). All possible orders of permutations (Pr) ∀ TS datasets in every cluster are tested. For *K* TS datasets in a cluster, there are *K*! possible permutations (e.g., a cluster with 5 datasets has 120 Pr for execution). Average dissimilarities between every TS dataset and other TS datasets in the cluster are indicated in Table 5. TS ‘a’ has the minimum average dissimilarity value (6.094). Because DTW is symmetric, dissimilarity is measured only for unique pairs. Minimum dissimilarity value in each cluster is indicated in Table 6. Using ClRe 1.0 (Younan et al., 2020a), dataset with label ‘a’ is selected as a cluster representative for clusters CLS_A, and CLS_B, while dataset ‘c’ is selected for clusters CLS_C and CLS_D.

**Table 4:** Datasets used for forming TS of the four clusters.

Dataset label	Items
a	[4, 5, 6, 19, 18, 5, 17, 14, 6, 11, 10, 8, 8, 9, 8, 8, 6, 5, 8, 9, 19, 23, 24, 18, 14, 23, 21]
b	[5, 8, 10, 7, 11, 13, 5, 4, 9, 10, 8, 8, 9, 8, 12, 16, 16, 14, 6, 8, 17, 13, 16, 18]
c	[6, 7, 5, 4, 4, 5, 6, 7, 8, 9, 9, 10, 5, 17, 13, 21, 26, 16, 18, 10, 9, 8, 7, 16, 17]
d	[4, 19, 18, 5, 14, 14, 16, 11, 10, 8, 8, 9, 8, 8, 6, 5, 8, 9, 19, 23, 24, 14, 25, 21, 24]
e	[7, 6, 5, 10, 8, 9, 11, 17, 18, 16, 15, 12, 10, 9, 21, 19, 19, 20, 7, 27, 22, 10, 11, 14]
f	[4, 5, 6, 19, 8, 5, 17, 14, 6, 11, 10, 8, 16, 9, 8, 18, 6, 5, 6, 17, 16, 24, 9, 10]

**Table 5:** Real example: dissimilarities between datasets and each other.

Dataset label	Items	a	b	c	d	e	f	Average dissimilarity
a	27	0	7.392	7.981	1.385	9.843	9.961	**6.094**
b	24		0	5.673	9.633	7.729	7.792	6.370
c	25			0	11.320	6.898	10.816	7.115
d	25				0	13.102	12.755	8.032
e	24					0	7.771	7.557
f	24						0	8.182

**Table 6:** Real example: selected representatives 8 clusters using ClRe 1.0.

Dataset label	Min.Dis.CLR_A	Min.Dis.CLR_B	Min.Dis.CLR_C	Min.Dis.CLR_D
a	–	4.189	5.320	6.094
b	4.355	–	–	–
c	–	–	–	–
d		–	–	–
e			–	–
f				–

Because cluster CLS_A has 3 datasets, its representatives in all permutations are the same in LE and PME, Thus it is excluded from the comparison. Table 7 presents summary for ClRe performance analysis running in LE mode on clusters CLS_B, CLS_C, and CLS_D. As mentioned previously, clusters are formed using real example datasets in Table 4. On the other hand, Table 8 presents summary for ClRe performance analysis running in PME mode on the same clusters of the real example (CLS_B : CLS_D). As indicated in Younan et al. (2020a), capturing almost of SThs behaviors in a cluster representative could decrease dissimilarity value between the cluster representative and other TS datasets in the cluster, on the other side, it may cause a slight increase in the cluster representative length.

**Table 7:** Real example: ClRe performance analysis in LE mode.

Cluster	Min.Dis	Alg.	Dissimilarity					Length				
			Min	Avg	50^*th*^ Perc	Max	Range	Min	Avg	50^*th*^ Perc	Max	Range
CLS_B	MinDs=a	ClRe 3.0	3.31	4.09	3.98	5.05	1.74	19	21	22	23	4
		ClRe 3.1	2.83	3.37	3.32	3.85	1.02	24	26	26	28	4
		ClRe 3.2	5.32	7.28	7.10	9.31	4.00	7	9	9	11	4
CLS_C	MinDs=a	ClRe 3.0	3.82	5.31	5.27	7.67	3.85	16	20	20	23	7
		ClRe 3.1	3.31	4.38	4.40	5.41	2.10	22	25	25	27	5
		ClRe 3.2	6.95	9.31	9.14	13.95	6.99	6	8	8	9	3
CLS_D	MinDs=a	ClRe 3.0	4.63	6.31	6.15	10.63	6.00	14	19	19	24	10
		ClRe 3.1	3.71	5.12	5.10	6.56	2.85	20	24	24	30	10
		ClRe 3.2	7.59	10.20	9.88	14.54	6.94	5	7	7	10	5

**Table 8:** Real example: ClRe performance analysis in PME mode.

Cluster	Min.Dis	Alg.	Dissimilarity					Length				
			Min	Avg	50^*th*^ Perc	Max	Range	Min	Avg	50^*th*^ Perc	Max	Range
CLS_B	MinDs=a	ClRe 3.0	3.27	3.56	3.43	3.97	0.70	19	21	21	23	4
		ClRe 3.1	2.78	2.99	3.05	3.14	0.36	24	26	26	27	3
		ClRe 3.2	6.65	8.16	8.78	9.03	2.38	7	8	8	9	2
CLS_C	MinDs=a	ClRe 3.0	4.22	5.71	5.50	9.97	5.74	16	18	17	23	7
		ClRe 3.1	3.32	4.09	4.01	4.91	1.59	22	25	25	28	6
		ClRe 3.2	7.46	9.75	8.80	12.52	5.06	6	8	8	9	3
CLS_D	MinDs=a	ClRe 3.0	5.21	6.52	6.18	9.69	4.48	15	17	17	20	5
		ClRe 3.1	4.14	4.90	4.88	5.83	1.69	21	24	24	28	7
		ClRe 3.2	8.38	10.42	10.66	14.04	5.66	6	7	8	9	3

### Real dataset

This subsection tests ClRe extensions using linear and pair-merge execution methods on four clusters formed using Szeged-weather dataset. This dataset contains real historical weather on temperature, pressure, wind speed and more. High level clustering is done on sensor type (phenomenon). In this experiment, temperature is selected as a higher level cluster; where annual cycles in temperature are extracted to represent datasets produced by similar sensors (i.e., lower level clustering). Clusters are formed using temperature readings on ‘January’ for years from 2011:2016 with TS labels from a:f as indicated in Table 9, dataset ‘b’ has the minimum average dissimilarity value (2.379). Thus, resulting clusters are CLS_A: {a:c}, CLS_B: {a:d}, CLS_C: {a:e}, and CLS_D: {a:f}. ClRe algorithms generate cluster representative for temperature cluster that hosts time series readings on January for years 2011:2016.

**Table 9:** Real dataset: dissimilarities between datasets and each other.

Dataset label	Items	a	b	c	d	e	f	Average dissimilarity
a	y_11=577	0	2.385	5.376	2.594	4.990	4.212	3.260
b	y_12=577		0	3.471	1.799	2.828	3.794	**2.379**
c	y_13=577			0	4.122	2.348	2.623	2.990
d	y_14=577				0	3.642	3.822	2.663
e	y_15=577					0	3.053	2.810
f	y_16=577						0	2.917

Similarly as discussed in the previous subsection, average dissimilarities between every dataset and other datasets in the cluster are indicated in Table 9. Minimum dissimilarity value in each cluster is indicated in Table 10. ClRe extensions are tested on four clusters as well in mode (Table 11) and in PME mode (Table 12). All permutations are tested to clarify the impact of sorting TS datasets by dissimilarity value on ClRe performance, and it is noticed that ClRe execution on ordered TS produces dissimilarity value ≈ the average dissimilarity ∀ permutations. For instance, for CLS B in real dataset experiments, ClRe 3.1 produces 1.54 in LE and 1.24 in PME mode (Tables 11 and 12).

**Table 10:** Real dataset: selected representatives ∀ clusters using ClRe 1.0.

Dataset label	Min.Dis.CLR_A	Min.Dis.CLR_B	Min.Dis.CLR_C	Min.Dis.CLR_D
a	–	–	–	–
b	1.952	1.914	2.097	2.379
c	–	–	–	–
d		–	–	–
e			–	–
f				–

**Table 11:** Real dataset: ClRe performance analysis in LE mode.

Cluster	Min.Dis	Alg.	Dissimilarity					Length				
			Min	Avg	50^*th*^ Perc.	Max	Range	Min	Avg	50^*th*^ Perc.	Max	Range
CLS_B	MinDs=b	ClRe 3.0	1.47	1.66	1.65	1.93	0.47	410	420	420	433	23
		ClRe 3.1	1.31	1.50	1.50	1.75	0.44	516	534	533	548	32
		ClRe 3.2	3.04	3.74	3.69	4.37	1.33	62	78	79	88	26
CLS_C	MinDs=b	ClRe 3.0	1.54	1.92	1.90	2.85	1.31	390	412	413	445	55
		ClRe 3.1	1.44	1.74	1.73	2.50	1.07	524	534	534	553	29
		ClRe 3.2	3.58	4.44	4.13	6.77	3.18	39	61	61	77	38
CLS_D	MinDs=b	ClRe 3.0	1.81	2.19	2.14	3.25	1.44	387	414	414	440	53
		ClRe 3.1	1.59	1.96	1.93	2.81	1.22	519	533	533	555	36
		ClRe 3.2	3.93	5.20	4.99	8.07	4.15	30	51	51	69	39

**Table 12:** Real dataset: ClRe performance analysis in PME mode.

Cluster	Min.Dis	Alg.	Dissimilarity					Length				
			Min	Avg	50^*th*^ Perc	Max	Range	Min	Avg	50^*th*^ Perc	Max	Range
CLS_B	MinDs=b	ClRe 3.0	1.30	1.34	1.32	1.41	0.11	532	550	553	564	32
		ClRe 3.1	1.20	1.24	1.24	1.30	0.10	638	651	654	662	24
		ClRe 3.2	3.50	3.79	3.81	4.06	0.56	70	74	73	78	8
CLS_C	MinDs=b	ClRe 3.0	1.33	1.58	1.57	1.95	0.62	470	518	516	550	80
		ClRe 3.1	1.26	1.48	1.46	1.72	0.46	621	657	655	686	65
		ClRe 3.2	3.67	4.04	3.92	5.66	1.99	44	65	64	81	37
CLS_D	MinDs=b	ClRe 3.0	1.49	1.67	1.67	1.80	0.31	538	593	593	626	88
		ClRe 3.1	1.42	1.56	1.57	1.68	0.26	704	751	754	781	77
		ClRe 3.2	4.14	4.69	4.70	5.10	0.96	47	56	56	69	22

**Figure 2:**
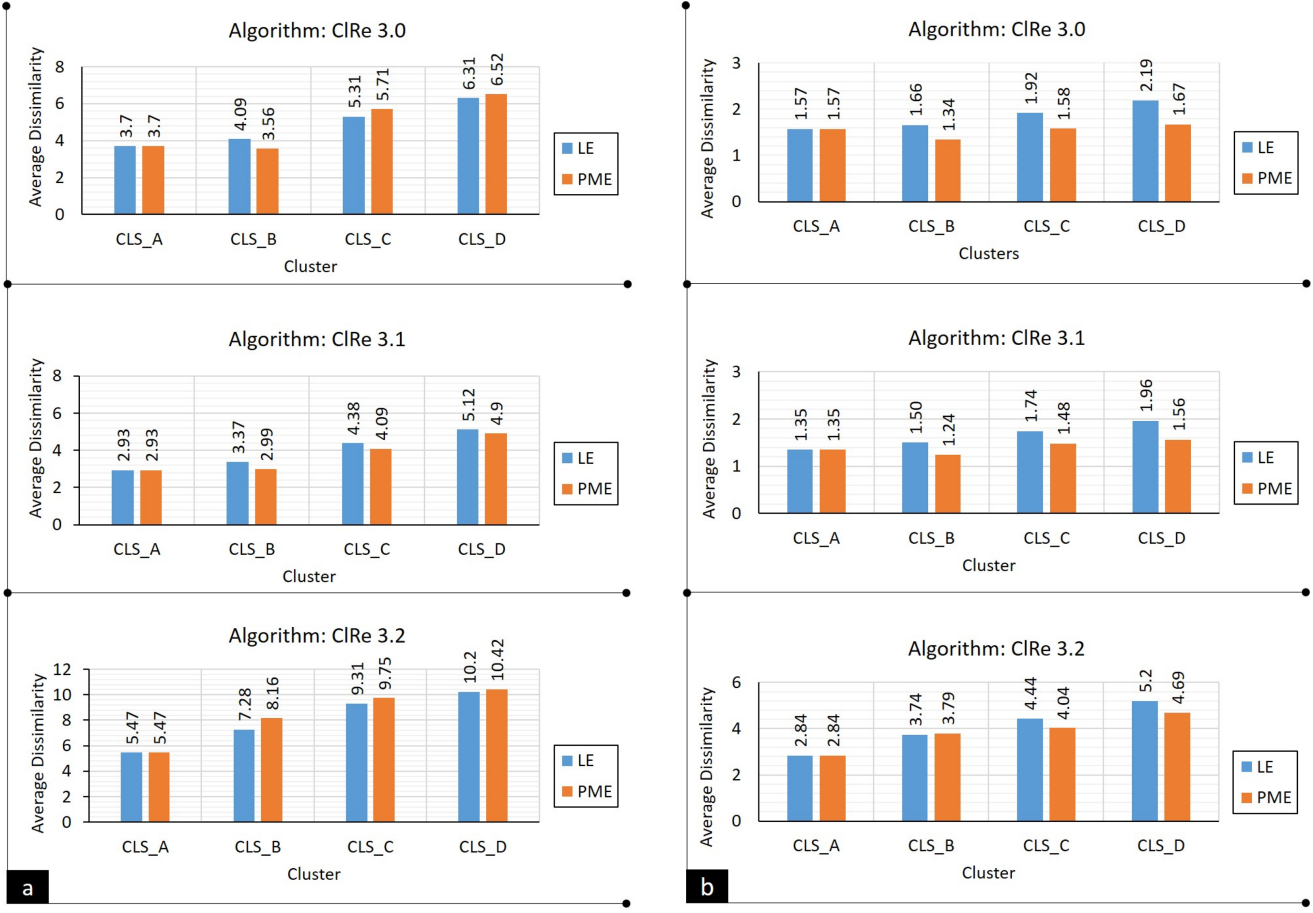
Real dataset: performance analysis for ClRe 3.0 and its extensions using LE and PME methods: (A) Real example, (B) real dataset.

To sum up, the proposed methods have an impact on the performance of ClRe 3.0 and its extensions (versions 3.1 and 3.2), Consequently, Tables 13 and 14 summarize and compare their average dissimilarity scores and resulting average datasets lengths using LE and PME methods. Figure 2 visualizes these results as well; where blue bars represent LE results, while red bars represent PME results. Evaluation performance for resulting representatives using LE and PME is measured using Eq. (4).

**Table 13:** Real example: a comparison for average dissimilarities and lengths of ClRe representatives in LE and PME modes.

Evaluation	Cluster	Linear			Pair Merge		
		ClRe 3.0	ClRe 3.1	ClRe 3.2	ClRe 3.0	ClRe 3.1	ClRe 3.2
Average dissimilarity	Ds=4	4.09	3.37	7.28	3.56	2.99	8.16
	Ds=5	5.31	4.38	9.31	5.71	4.09	9.75
	Ds=6	6.31	5.12	10.2	6.52	4.9	10.42
Average length	Ds=4	21	26	9	21	26	8
	Ds=5	20	25	8	18	25	8
	Ds=6	19	24	7	17	24	7

**Table 14:** Real dataset: a comparison for average dissimilarities and lengths of ClRe representatives in LE and PME modes.

Evaluation	Cluster	Linear			Pair Merge		
		ClRe 3.0	ClRe 3.1	ClRe 3.2	ClRe 3.0	ClRe 3.1	ClRe 3.2
Average dissimilarity	Ds=4	1.66	1.50	3.74	1.34	1.24	3.79
	Ds=5	1.92	1.74	4.44	1.58	1.48	4.04
	Ds=6	2.19	1.96	5.2	1.67	1.56	4.69
Average length	Ds=4	420	534	78	550	651	74
	Ds=5	412	534	61	518	657	65
	Ds=6	414	533	51	593	751	56

(4) $$Enhancement(ClRe)=\left(1-\frac{Dis_{PME}}{Dis_{LE}}\right)\times 100$$

where *Dis*_*PME*_ and *Dis*_*LE*_ are dissimilarity values in case of running ClRe algorithm in PME and LE modes respectively. Based on this equation, evaluation results of real dataset experiments indicate that PME could improve performance of ClRe versions 3.0, 3.1, and 3.2 in average by approximately 20.5%, 17.7%, and 6.4%, respectively.

## Conclusion

As SThs penetrate our life, the future IoT has to provide services that enable interoperability between SThs for reducing SThs integration, on the other hand data production in IoT application has to be balanced in terms of accuracy, volume, and search for presenting meaningful information for IoT users (machines and human). Clustering resulting big IoT data and removing redundancy by capturing most of SThs behaviors still requires deep research. A novel data reduction model is proposed in the previous work and improved in this paper to index clusters representatives with higher accuracy (i.e., captures and expresses most of SThs behaviors). Data reduction model on the SThs level for data gathering is recommended to be improved by implementing matrix completion. Complementary data fusion still not addressed in this work and could present a promising solution for indexing entities of interest (e.g., room state), where their states rely on correlation between set of SThs.

## Supplemental Information

10.7717/peerj-cs.500/supp-1 Supplemental Information 1 YouRe Execution Methods Code written in Python using Jupyter.

## Additional Information and Declarations

### Data Availability

The following information was supplied regarding data availability: The time series data is available at Kaggle: https://www.kaggle.com/budincsevity/szeged-weather. The code (ClRe Execution Methods) is available at GitHub: https://github.com/MinaYounan-CS/ClRe.
